# Cost-Effectiveness of Pediatric Central Venous Catheters in the UK: A Secondary Publication from the CATCH Clinical Trial

**DOI:** 10.3389/fphar.2017.00644

**Published:** 2017-09-19

**Authors:** Colin H. Ridyard, Catrin O. Plumpton, Ruth E. Gilbert, Dyfrig A. Hughes

**Affiliations:** ^1^Centre for Health Economics and Medicines Evaluation, Bangor Institute for Health and Medical Research, Bangor University Bangor, United Kingdom; ^2^UCL Institute of Child Health, University College London London, United Kingdom

**Keywords:** cost-effectiveness analysis, bloodstream infection, central venous catheter, pediatric intensive care, antibiotic, heparin

## Abstract

**Background:** Antibiotic-impregnated central venous catheters (CVCs) reduce the risk of bloodstream infections (BSIs) in patients treated in pediatric intensive care units (PICUs). However, it is unclear if they are cost-effective from the perspective of the National Health Service (NHS) in the UK.

**Methods:** Economic evaluation alongside the CATCH trial (ISRCTN34884569) to estimate the incremental cost effectiveness ratio (ICER) of antibiotic-impregnated (rifampicin and minocycline), heparin-bonded and standard polyurethane CVCs. The 6-month costs of CVCs and hospital admissions and visits were determined from administrative hospital data and case report forms.

**Results:** BSIs were detected in 3.59% (18/502) of patients randomized to standard, 1.44% (7/486) to antibiotic and 3.42% (17/497) to heparin CVCs. Lengths of hospital stay did not differ between intervention groups. Total mean costs (95% confidence interval) were: £45,663 (£41,647–£50,009) for antibiotic, £42,065 (£38,322–£46,110) for heparin, and £44,503 (£40,619–£48,666) for standard CVCs. As heparin CVCs were not clinically effective at reducing BSI rate compared to standard CVCs, they were considered not to be cost-effective. The ICER for antibiotic vs. standard CVCs, of £54,057 per BSI avoided, was sensitive to the analytical time horizon.

**Conclusions:** Substituting standard CVCs for antibiotic CVCs in PICUs will result in reduced occurrence of BSI but there is uncertainty as to whether this would be a cost-effective strategy for the NHS.

## Introduction

Central venous catheters (CVCs) are a large yet potentially avoidable cause of health-care associated infections in hospitals. In pediatric intensive care units (PICUs), catheter-related bloodstream infections (BSIs) occur in 3–8% of all CVC insertions (Hockenhull et al., 2008). BSIs are associated with increased morbidity, mortality, lengths of hospital stay, and healthcare costs (Abou Elella et al., 2010; Nowak et al., 2010). Since between 40 and 60% (Harron et al., 2014) of the 16,000 annual admissions to English PICUs (PICANet, 2014) require CVCs, BSIs represent a major burden to patients and the National Health Service (NHS) (Elward et al., 2005; Abou Elella et al., 2010).

The incidence of BSI in adults may be reduced with CVCs impregnated with antibiotics, antibacterial agents or heparin. These are recommended for use in adults at highest risk of BSI (Department of Health, 2007), but evidence in children is lacking (Balain et al., 2015). CVC use in children presents a greater theoretical risk of BSI owing to the narrower lumens within which blood may thrombose more readily. The CATheter Infections in Children (CATCH) trial (NCT01029717) was a pragmatic, three-arm randomized controlled trial aimed to determine the clinical and cost-effectiveness of antibiotic or heparin CVCs compared with standard CVCs in children requiring intensive care. Both heparin-bonded and antibiotic-impregnated CVCs prevent biofilm formation which prevents bacterial colonization. Heparin inhibits thrombus formation and heparin-bonded CVCs use benzalkonium chloride as an anti-infective bonding agent. The primary analyses of CATCH, however, showed no effect of impregnated compared with standard CVCs (Gilbert et al., 2016; Harron et al., 2016a); but secondary analyses revealed antibiotic CVCs to be superior to heparin CVCs with a hazard ratio (HR) for time to first BSI of 0.42 (95% CI, 0.19–0.93), and to standard polyurethane CVCs (HR 0.43; 95% CI 0.20–0.96). Heparin CVCs were no different from standard (HR 1.04; 95% CI, 0.53–2.03).

As impregnated CVCs are more expensive than standard, decisions on their broader use within the NHS requires evidence of their cost-effectiveness. Existing economic analyses are limited in their applicability to the PICU setting in the UK as they relate to adult populations and, with one exception (Hockenhull et al., 2008), apply to different healthcare systems [Australia (Halton et al., 2009), Germany (Frank et al., 2003), and the USA (Veenstra et al., 1999; Marciante et al., 2003; Shorr et al., 2003)]. These studies indicate, however, that antibiotic-impregnated CVCs are associated with improved health outcomes and are cost saving.

Previous economic evaluations are reliant on modeled costs and consequences of BSI using data from a range sources, often observational studies. As such, they rely on assumed attribution of hospital lengths of stay (the main cost driver) and mortality to BSI. The economic evaluation which adopted an NHS cost perspective assumed catheter-related BSIs increase the length of hospital stay by 6 additional days in intensive care units (ICU) and 5 additional days in a general medical ward (Hockenhull et al., 2008). A US cohort study of 1,339 pediatric cases of catheter-related BSI matched to controls by propensity-score, identified a higher mean attributable length of stay of 19 days (Goudie et al., 2014). While this is comparable with the 21 days excess length of stay estimated for BSI in pediatric hematology/oncology patients (Wilson et al., 2014), studies of this nature are based on retrospective observational data and are prone to bias. Patients who are more ill are more likely to develop BSI, making it difficult to separate the contribution of BSI to excess length of stay from the underlying condition.

The aim of the present study was to assess the cost-effectiveness of antibiotic and heparin CVCs relative to commonly used standard polyurethane CVCs in a UK PICU setting using data collected as part of the CATCH randomized controlled trial.

## Methods

### Design and results of CATCH

CATCH recruited 1,485 children <16 years who were admitted to any of 14 PICUs in England and who were expected to require a CVC for ≥3 days. Children were randomized equally to receive heparin-bonded, antibiotic-impregnated (rifampicin and minocycline), or standard polyurethane CVCs. The intervention was blinded to everyone except the clinicians responsible for inserting the catheter. The primary outcome was the time to first BSI occurring between 48 h after randomization and 48 h after CVC removal. This occurred in 3.59% (18/502) children randomized to standard CVC, 1.44% (7/486) to antibiotic, and 3.42% (17/497) to heparin CVCs. In the primary analysis, impregnated CVCs (antibiotic and heparin) were no more effective than standard CVCs (HR 0.71; 95% CI 0.37–1.34). Antibiotic CVCs were superior to standard CVCs in secondary analysis (HR 0.43; 0.20–0.96) but heparin CVCs were not (HR 1.04; 0.53–2.03). There were no differences between intervention groups in other outcomes, including time to thrombosis, 30-day mortality, or antibiotic resistance (minocycline or rifampicin). Trial results are presented in full elsewhere (Gilbert et al., 2016; Harron et al., 2016a).

The CATCH trial is registered with the ClinicalTrials.gov (Trial registration: NCT01029717 Registered 9 December 2009), and was conducted in accordance with the recommendations of the Research Ethics Committee for South West England, with prospective or deferred written informed consent obtained from all subjects in accordance with the Declaration of Helsinki. The protocol was approved by the Research Ethics Committee for South West England (reference number 09/H0206/69), and is available at www.nets.nihr.ac.uk/projects/hta/081347.

### Economic evaluation

We conducted a cost-effectiveness analysis as it is not possible to estimate health utilities in children in a PICU setting (Thorrington and Eames, 2015). While this precluded any evidence on allocative efficiency, it allowed for an assessment of technical efficiency for selecting the most cost-effective CVC for reducing the occurrence of BSIs.

### Resource use

The economic analysis adopted the perspective of the NHS in England, with resource use measurement focused on the principal cost drivers, which were PICU, High Dependency Unit (HDU) and ward stays (including readmissions), outpatient clinic visits, Accident and Emergency (A&E) admissions and the costs of the CVCs. The 6-month time horizon of the base-case analysis was chosen to include the costs associated with managing BSIs and associated complications. Shorter time horizons were explored in sensitivity analyses.

Patients' use of hospital services were obtained from trial case report forms (CRF), Hospital Episode Statistics (HES), the Paediatric Intensive Care Audit Network (PICANet), and hospital Patient Administration Systems (PAS). CRFs were accessed for data on dates of hospital discharge, transfer to other hospitals and CVC removal. HES data on Healthcare Resource Groups (HRGs) corresponding to the type of care patients receive at a ward-level, outpatient visits and A&E admissions, were accessed from NHS Digital^1^. We accessed the PICANet dataset^2^ for the National Schedule of Reference Cost HRGs for HDU and ICU stays^3^, and for verifying the dates of hospital admission, transfer and discharge. The finance offices of each participating hospital provided data from Patient Administration Systems (PAS) on patients' lengths of stay in ICUs and wards, and on relevant HRGs. These were used to supplement data that were otherwise missing from other sources.

### Costs analysis

HRGs reflect NHS hospital payments for patients' use of hospital services. Unit costs from the 2012 to 2013 National Schedule of Reference Costs were applied to all HRG codes; the most significant being those associated with PICU, Neonatal Intensive Care Unit (NICU) and HDU (Table 1). Basic HDU (XB07Z) or ICU (XB05Z) codes were applied in the 10% of cases where HRG codes were missing.

**Table 1:** Unit cost for intensive care and high dependency care, based on HRGs from the National Schedule of Reference Costs (2012–13).

**HRG code**	**HRG name**	**Description**		**Cost per day (£)**
XB01Z	Pediatric critical care, intensive care, ECMO/ECLS	Highly specialized intensive care treatment	ECMO, VAD, and other highly complex procedures	4,391
XB02Z	Pediatric critical care, intensive care, advanced enhanced		Unstable multi-system failure with other complications	2,409
XB03Z	Pediatric critical care, intensive care, advanced	Intensive nursing supervision at all times, undergoing complex monitoring and/or therapeutic procedures, including advanced respiratory support	Invasive ventilation with multi-system failure	2,017
XB04Z	Pediatric critical care, intensive care, basic enhanced		Intensive ventilation with more than one system failure	2,110
XB05Z	Pediatric critical care, intensive care, basic	Continuous nursing supervision	Invasive ventilation with single system failure *or* non-invasive ventilation with more than one system failure	1,743
XB06Z	Pediatric critical care, high dependency, advanced	Require closer observation and monitoring than is usually available on an ordinary children's ward, with higher than usual staffing levels	Non-invasive ventilation (e.g., CPAP and BiPAP by mask with IV drugs)	1,335
XB07Z	Pediatric critical care, high dependency		Close monitoring, oxygen by mask, no invasive ventilation)	886
XB08Z	Pediatric critical care, transportation	Since pediatric critical care facilities are centralized in a small number of hospitals providing expert specialist care, specialist transport teams are required to deliver clinical management during transfer of patients		2,799
XA01Z	Neonatal critical care, intensive care	Care provided for babies who are the most unwell or unstable and have the greatest needs in relation to staff skills and staff to patient ratios	Baby receives any form of mechanical respiratory support via a tracheal tube and/or parenteral nutrition	1,118

*ECMO, extra-corporeal membrane oxygenization; ECLS, extracorporeal life support; VAD, Ventricular assist devices; CPAP, Continuous Positive Airway Pressure; BiPAP, Bi-Level Positive Air Pressure; IV, intravenous*.

Unit costs of ward, outpatient and A&E attendances are presented in the Supplementary Appendix Tables 1–3. Any missing HRGs from HES or PAS data were replaced with ward costs based on bed-day rates provided by hospital finance offices (Supplementary Appendix Table 4). Bed-day rates were also applied to unassignable HRG codes appearing in the HES and PAS data, but overall, bed-day rates were used to cost <1% of admissions.

Catheter list prices were provided by the supplier (Cook Medical, Bloomington, IN, USA).

The costs of care for the 6-months prior to randomization were calculated from HES and PICANet data. Given that HRGs relate to episodes of care, we calculated patient costs for the 6-months following randomization according to:

$$\begin{array}{l} \text{Cost}=(\text{N}/\text{n}+\text{N})\times (\text{wardcost}+\text{PICUcost}+\text{HDUcost})\\ \;+\;(\text{outpatient costs}+\text{A}\&\text{E costs}+\text{CVC}\;\text{costs}) \end{array}$$

Where n and N are the number of days patients were hospitalized prior to, and following randomization, respectively.

Patients' use of healthcare resources and total costs were calculated for the intention to treat population, and summary statistics were generated by intervention group.

### Outcomes

The health outcome for the cost-effectiveness analysis was the presence of a first BSI. These were defined in CATCH by a positive blood culture from a sample that was clinically indicated and taken more than 48 h after CVC insertion and up to 48 h after CVC removal (Gilbert et al., 2016).

### Incremental analysis

Each CVC was ranked in order of decreasing effectiveness and dominated interventions (those which are less effective or ineffective) or extendedly dominated interventions eliminated. The incremental cost effectiveness ratio (ICER) was calculated for remaining CVCs as the difference in the means of total costs divided by the difference in the proportion of bloodstream infections.

### Uncertainty analysis

Bias-adjusted 95% central ranges for differences in costs and BSI were calculated from 10,000 replicate bootstrap analyses. The joint uncertainty in costs and BSI was depicted in a cost-effectiveness acceptability curve (CEAC) which presented the probability of CVCs being cost effective for different threshold willingness to pay for each BSI averted (Fenwick et al., 2001).

Uncertainty in total costs was further assessed by adjusting for the contribution of baseline factors to overall variability (Mihaylova et al., 2011).

### Sensitivity analysis

Given the dependency of costs and therefore the ICER on the analytic time horizon, a sensitivity analysis was performed in which costs were limited to those incurred during the index hospitalization (that is, excluding any re-admissions that may have occurred over the 6-month period).

### Regression analysis

Regression analyses were performed to control for possible baseline imbalances between intervention groups (Mihaylova et al., 2011) and, by including a variable to representing the presence of a BSI, to estimate the value of healthcare resources associated with the management of BSI. The following pre-specified variables were tested for their independent associations with total costs: Age, body weight, 6-month pre-randomization costs (all log-transformed), gender, pre-existing CVC 72 h prior to randomization, health status before PICU admission, reason for admission (cardiovascular, endocrine or metabolic, infection, neurological, oncology, respiratory, trauma, other), suspected infection at randomization, immune compromised, positive blood culture within 72 h prior to randomization, numbers of devices *in situ*, and admission type (elective or emergency). Where data were missing, we assumed: patients to be healthy (*n* = 1), not immunocompromised (*n* = 19), and no positive blood culture (*n* = 5). Missing data for weight (*n* = 2) were imputed with the mean (11.95 kg).

Variables that were significant at the 5% level were included using a stepwise approach in multivariable generalized linear models (GLMs) that were specified using a combination of families (e.g., gamma and poisson) and links (e.g., log, square root and identity). Modified Park's test and Akaike Information Criterion were used to assess GLM goodness of fit but were inconclusive. The identity link function performed best according to the Pearson Correlation, Pregibon Link, and the Modified Hosmer and Lemeshow tests. We therefore specified an ordinary least squares regression based on the comparatively large sample size which guaranteed near-normality of sample means (Glick et al., 2007).

All analyses were performed using STATA Version 10, and the economic analysis was reported according to the Consolidated Health Economic Evaluation Reporting Standards (CHEERS) statement (Husereau et al., 2013).

## Results

### Resource use and costs

Cost data were available for all patients. Hospital ICU/HDU length of stay and total costs were comparable between intervention groups during the 6-months prior to randomization.

In the 6-months following randomization, patients randomized to antibiotic-impregnated CVCs were in PICU for a mean of 10.8 days (95% CI, 9.3–12.4), compared with 9.9 days (95% CI, 8.6–11.4) for those randomized to heparin-bonded CVC and 10.5 days (95% CI, 9.2–11.9) for standard CVCs (Table 2). Mean durations of hospitalization were 34.8 days (95% CI, 31.2–38.5) for antibiotic-impregnated CVC, 31.4 days (95% CI, 28.2–34.7) for heparin-bonded CVC and 31.7 (95% CI, 28.8–34.7) for the standard CVC group. Six HRGs (from a total of 349) relating to congenital or other cardiac surgery and lower respiratory tract disorders, accounted for 50% of ward costs.

**Table 2:** Patients' lengths of stay from randomization to 6-months (including readmissions), according to place and intensity of care and by intervention group.

**Unit**	**Antibiotic CVC**		**Heparin CVC**		**Standard CVC**	
	**Mean**	**95% CI**	**Mean**	**95% CI**	**Mean**	**95% CI**
Days on ICU	10.79	9.28, 12.48	9.91	8.57, 11.44	10.50	9.17, 11.93
Pediatric critical care, intensive care, ECMO/ECLS	0.30	0.07, 0.72	0.38	0.09, 0.80	0.40	0.17, 0.72
(XB01Z)						
Pediatric critical care, intensive care, advanced	0.16	0.09, 0.26	0.12	0.09, 0.15	0.16	0.10, 0.26
enhanced (XB02Z)						
Pediatric critical care, intensive care, advanced (XB03Z)	0.76	0.51, 1.05	0.61	0.43, 0.83	0.65	0.46, 0.87
Pediatric critical care, intensive care, basic enhanced	2.30	1.92, 2.72	2.68	2.09, 3.44	2.75	2.14, 3.54
(XB04Z)						
Pediatric critical care, intensive care, basic (XB05Z)	6.96	5.65, 8.45	5.63	4.75, 6.59	6.40	5.42, 7.47
Neonatal critical care, intensive care (XA01C)	0.29	0.10, 0.55	0.46	0.13. 1.03	0.11	0.04, 0.20
Days on HDU	1.99	1.48, 2.62	1.59	1.28, 1.99	1.73	1.44, 2.05
Pediatric critical care, high dependency, advanced	1.27	0.94, 1.70	1.08	0.80, 1.45	1.22	0.98, 1.49
(XB06Z)						
Pediatric critical care, high dependency (XB07Z)	0.71	0.42, 1.16	0.51	0.40, 0.64	0.51	0.40, 0.64
Days on ward	22.01	19.26, 24.80	19.84	17.40, 22.40	19.48	17.12, 21.94
Total days in hospital	34.80	31.21, 38.48	31.35	28.18, 34.65	31.71	28.75, 34.81
Count of non-PICU/HDU inpatient HRGs						
Complex congenital surgery (EA24Z)	100		103		109	
Intermediate congenital surgery (EA25Z)	68		70		72	
Major complex congenital surgery (EA23Z)	45		39		37	
Cardiac conditions with complication and comorbidity	109		102		74	
(PA23A)						
Lower Respiratory Tract Disorders without acute	95		78		105	
bronchiolitis with length of stay ≥1 day with complication						
and comorbidity (PA14C)						
Implantation of prosthetic heart or ventricular assist	2		2		4	
device (EA43Z)						
Other inpatient HRGs	1,103		1,055		964	

*CVC, central venous catheter; CI, confidence interval; ICU, Intensive care unit; ECMO, extra-corporeal membrane oxygenization; ECLS, extracorporeal life support; HDU, High dependence unit; PICU, Pediatric intensive care unit; HRGs, Healthcare Resource Groups*.

Mean 6-month costs were £44,503 (median £28,952; range £1,786–£360,983; 95% CI, £40,619–£48,666) for standard CVC, £45,663 (median £29,793; range £2,189–£442,365; 95% CI, £41,647–£50,009) for antibiotic-impregnated CVC, £42,065 (median £27,621; range £2,638–£382,431; 95% CI, £38,322–£46,110) for heparin-bonded CVC (Table 3). Costs were not significantly different by CVC group over the 6-month timeframe.

**Table 3:** Disaggregated and total costs (£) by intervention group from randomization to end of the 6-month timeframe.

**Unit (code)**	**Antibiotic CVC**		**Heparin CVC**		**Standard CVC**	
	**Mean**	**95% CI**	**Mean**	**95% CI**	**Mean**	**95% CI**
Pediatric Critical Care, Intensive Care						
ECMO/ECLS (XB01Z)	1,358	310, 3,159	1,703	386, 3,509	1,796	723, 3,156
Advanced enhanced (XB02Z)	388	207, 636	289	216, 371	395	228, 620
Advanced (XB03Z)	1,545	1,031, 2,124	1,250	872, 1,674	1,318	933, 1,752
Basic enhanced (XB04Z)	4,861	4,060, 5,738	5,675	4,418, 7,260	5,822	4,512, 7,460
Basic (XB05Z)	12,137	9,855, 14,730	9,822	8,274, 11,489	11,159	9,440, 13,025
Neonatal critical care, intensive care (XA01C)	325	113, 613	517	142, 1,150	125	42, 225
Pediatric Critical Care, HDU						
High dependency, Advanced (XB06Z)	1,709	1,254, 2,271	1,450	1,972, 1,940	1,629	1,301, 1,992
High dependency (XB07Z)	635	372, 1,025	454	354, 567	456	356, 566
Transportation (XB08Z)	1,158	1,022, 1,293	1,258	1,109, 1,413	1,208	1,068, 1,353
Sub-total (PICU/HDU/NICU)^a^	24,115	20,824, 27,764	22,417	19,429, 25,771	23,907	20,989, 27,049
Inpatient Stay^b^						
Complex congenital surgery (EA24Z)	3,011	2,445, 3,593	2,908	2,363, 3,481	3,144	2,565, 3,753
Intermediate congenital surgery (EA25Z)	2,166	1,670, 2,699	1,934	1,470, 2,440	2,044	1,583, 2,545
Major complex congenital surgery (EA23Z)	1,865	1,315, 2,481	1,915	1,310, 2,603	1,466	1,013, 1,960
Cardiac Conditions with complication and comorbidity	1,277	818, 1,845	1,173	831, 1,558	739	495, 1,025
(PA23A)						
Lower respiratory tract disorders without acute	858	593, 1,157	668	454, 913	943	657, 1,268
bronchiolitis with length of stay ≥1 day with complication						
and comorbidity (PA14C)						
Implantation of prosthetic heart or ventricular assist	273	0, 684	298	0,762	548	103, 1,155
device (EA43Z)						
Other inpatient HRG costs	10,316	8,616, 12,231	8,803	7,524, 10,106	9,930	7,860, 12,409
Sub-total (inpatient)	19,766	17,934, 21,755	17,700	16,308, 19,182	18,814	16,649, 21,327
A&E cost	89	76, 104	85	73, 99	91	78, 104
Outpatient cost	1,615	1,412, 1,838	1,784	1,496, 2,109	1,648	1,453, 1,871
CVC cost	78	78, 78	78	78, 78	43	43, 43
Total cost (full 6 months)	45,663	41,647, 50,009	42,065	38,322, 46,110	44,503	40,619, 48,666

a National Schedule of Reference Costs 2012–2013;

b *Top 6 (of 349) HRGs ranked by cost, together contributing 50% of overall inpatient cost, <1% taken from bed day rates*.

*CVC, central venous catheter; CI, confidence interval; ECMO, extra-corporeal membrane oxygenization; ECLS, extracorporeal life support; HDU, High dependence unit; PICU, Pediatric intensive care unit; NICU, Neonatal intensive care unit; HRGs, Healthcare Resource Groups; A&E, Accident and Emergency*.

Variables tested for the cost regression were evenly balanced between intervention groups (Balain et al., 2015). The residual variability in total cost could be explained, in part, by the following significant explanatory variables: age (in days), 6-month pre-randomization costs (both log-transformed), health status at randomization, reason for admission, immune status, and admission type (elective or emergency). The adjusted incremental costs associated with antibiotic CVCs, in relation to standard CVCs, were £1,220 (95% CI, −£4,332 to £6,773), and with heparin CVCs, −£2,399 (95% CI, to −£7,914 to £3,120).

### Outcomes

Seven patients from 486 randomized to antibiotic CVCs experienced a BSI, compared with 17/497 in the heparin CVC group and 18/502 in the standard CVC group. A statistically significant absolute risk differences was found only for antibiotic vs. standard CVCs (−2.15%; 95% CI, −4.09 to −0.20). Heparin CVCs were not clinically effective with a risk difference of −0.17% (95% CI, −2.45 to 2.12) vs. standard CVC.

### Value of healthcare resources associated with BSI

Patients who had a BSI (*n* = 42) experienced 6.5 more days (95% CI, 1.4–11.6) in PICU than those with no BSI (*n* = 1,443), and 15.1 additional total days (95% CI, 4.0–26.2) of hospitalization. The mean 6-month costs for patients with a BSI was £60,481 (95% CI, £47,873–£73,809) compared to £43,578 (95% CI, £41,185–£45,970) for those without; a difference of £17,263 (95% CI, −£3,076 to £31,450). The adjusted difference in mean costs was £10,975 (95% CI, −£2,801 to £24,751).

### Incremental and uncertainty analysis

Heparin CVCs were not clinically effective when compared to standard CVC, and are more expensive, and so cannot be cost-effective by the same measure of BSI. The ICER for antibiotic-impregnated vs. standard CVCs was £54,057 per BSI averted (Table 4).

**Table 4:** Incremental analysis.

	**Antibiotic CVC**	**Heparin CVC**	**Standard CVC**
**BASE-CASE ANALYSIS (6-MONTH TIME HORIZON)**			
Total costs	£45,663 (£41,647, £50,009)	£42,065 (£38,322, £46,110)	£44,503 (£40,619, £48,666)
Incremental cost (vs. standard)	£1,160 (−£4,743, £6,692)	−£2,438 (−£8,164, £3,359)	–
BSI	1.44% (0.4, 2.5)	3.42% (1.8, 5.0)	3.59% (2.0, 5.2)
Incremental BSI (vs. standard)	−2.15% (−4.1, −0.2)	−0.17% (−2.5, 2.1)	–
ICER (vs. standard)	£54,057 per BSI averted	–^a^	–
**SENSITIVITY ANALYSIS (INDEX HOSPITALIZATION)**			
Total costs	£33,073 (£30,047, £36,337)	£32,245 (£29,013, £35,823)	£35,165 (£31,864, £38,670)
Incremental cost (vs. standard)	−£2,093 (−£6,919, £2,583)	−£2,920 (-£7,833, £2,180)	−
BSI	1.44% (0.4, 2.5)	3.42% (1.8, 5.0)	3.59% (2.0, 5.2)
Incremental BSI (vs. standard)	−2.15% (−4.1, −0.2)	−0.17% (−2.5, 2.1)	−
ICER (vs. standard)	−£97,543 per BSI averted^b^	−^a^	−

*Values are means with 95% confidence intervals in parentheses*.

a *As heparin CVC was not deemed to be clinically effective in reducing BSI rates, it cannot be cost-effective by the same outcome measure*.

*BSI, bloodstream infection; ICER, incremental cost effectiveness ratio; CVC, central venous catheter*.

b *Cost saving*.

**Figure 1:**
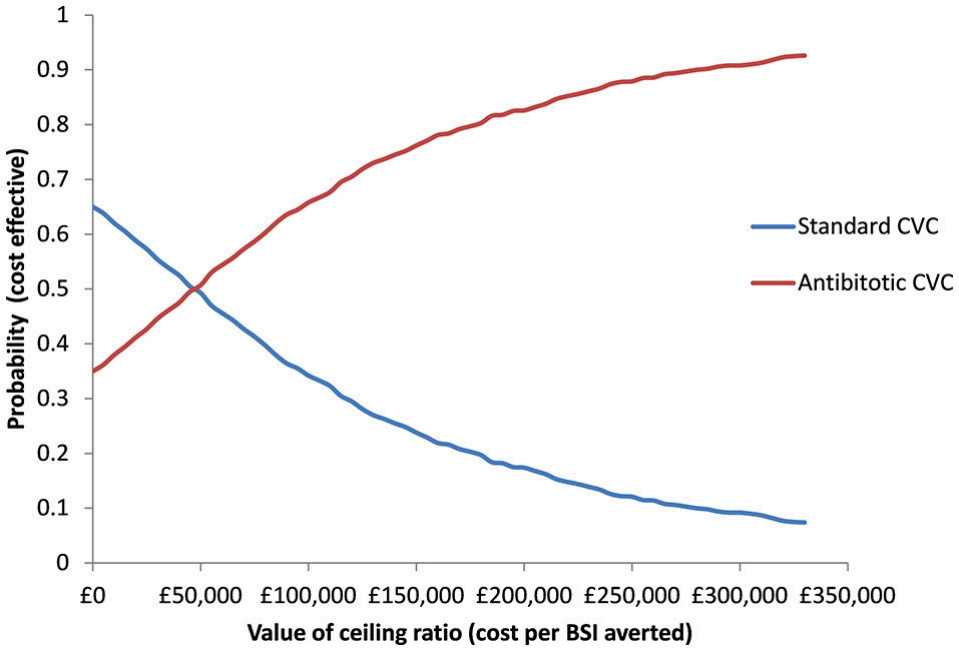
Cost-effectiveness acceptability curve presenting the probability of antibiotic and standard CVCs being cost-effective for a given values of ceiling ratio expressed as cost per bloodstream infection (BSI) averted.

The probabilities of antibiotic CVCs being cost-effective at thresholds of £10,000, £50,000, and £100,000 per BSI averted, were 0.38, 0.49, and 0.62, respectively (Figure 1). There is a probability of 0.650 for standard CVCs dominating antibiotic CVCs.

### Sensitivity analysis

Considering only the index hospitalization, total costs in the antibiotic CVC group were £33,073 (95% CI, £30,047–£36,337) compared to £32,245 (95% CI, £29,013–£35,823) in the heparin CVC group and £35,165 (95% CI, £31,864–£38,670) in the standard CVC group. Antibiotic CVCs therefore dominated standard CVC with a difference of 2.15% in the risk of BSI, and a saving of £97,543 per BSI averted.

## Discussion

The results of the base-case analysis indicate that heparin-bonded CVCs are not cost-effective while the incremental cost-effectiveness ratio of antibiotic-impregnated CVCs vs. standard CVCs is £54,057 per BSI averted. However, there is considerable uncertainty in this estimate. Restricting costs to the index hospital stay resulted in an ICER of £97,543 saved per BSI averted for antibiotic compared to standard CVCs. Antibiotic CVCs are highly cost-effective when considering costs accruing over comparable periods to events.

The economic analysis benefits from having been designed and executed as an integral part of a pragmatic clinical trial that provided an unbiased comparison of CVCs in the context of current practice in 14 UK PICUs. The cost-effectiveness analysis was conducted to accepted methodological standards of trial-based economic evaluations (Ramsey et al., 2015). Patient-level HES data were used to reflect NHS payments to hospitals for their services, and we exploited different sources to ensure a complete dataset.

However, there are limitations to the analysis. First, the CATCH trial was not powered to demonstrate statistically significant differences in effectiveness or costs among each of the three types of CVCs. However, differences in the rates of BSI were pre-specified in a secondary analysis, and a lack of a difference in costs between intervention groups is less relevant in the context of net benefits (Claxton, 1999). The joint uncertainty in costs and BSI is considered in the CEAC which indicated antibiotic CVCs as having a probability of 0.35 of dominating standard CVCs. Despite not being effective at reducing BSI rates, the mean costs associated with heparin CVCs were lower than for either antibiotic or standard CVCs. This is likely to be explained by BSI being a rare event, with associated costs diluted in the overall costs of managing patients in intensive care.

A second limitation was in our choice of economic outcome. The quality-adjusted life-year (QALY), which is the preferred measure of health outcome for cost-utility analyses (National Institute for Health and Care Excellence, 2013), could not be estimated in the study population (Thorrington and Eames, 2015). The majority of trial participants (58%) were aged <1 year, and even if utilities were measured by proxy, these would be unreliable, especially in the context of intensive care. Using BSI averted as the denominator of the ICER calculation also fails to fully capture other possible consequences of BSI, including long term neurological defects, mortality, antibiotic resistance (Falagas et al., 2007) and other adverse events (Tsai et al., 2016). While neurological outcomes were not monitored in CATCH, there were no differences in 30-day mortality for antibiotic vs. standard (HR 0.96; 95% CI, 0.61–1.51) or for heparin vs. standard CVC (HR 0.65; 95% CI, 0.40–1.07). There were also no differences between intervention groups in microbial resistance to minocycline or rifampicin, or in adverse event rates (Gilbert et al., 2016; Harron et al., 2016a).

In contrast to QALYs, where an explicit threshold range has been defined (£20,000–£30,000 per QALY gained for most health technologies in the UK), there is no threshold for BSIs averted. Interpretation may therefore be dependent on previous economic evaluations, such as Shorr et al. (2003) who considered US$9600 to be cost-effective, or assumptions concerning the impact of BSI on health. For instance, if BSIs are assumed to impair patients' quality of life by a year, (i.e., 1 QALY decrement on average), then antibiotic CVCs may not be cost-effective.

The choice of analytical time horizon represents a further limitation. Six months was selected to capture the costs of subsequent hospital readmissions and transfers to other hospitals. However, as the cost-effectiveness calculation considered only the first BSI, costs accrued over time with no corresponding change to the number of BSI (these all occurred within 30 days). Consequently, the ICER continued to increase over time.

Our estimates of the costs associated with the management of BSI are broadly in line with other economic evaluations (Hockenhull et al., 2008); however there are appreciable differences in our estimate of the ICER. Previous economic analyses indicated the dominance of antibiotic CVCs over standard CVCs. Possible explanations for this discrepancy are that model-based analyses are based on a synthesis of data from disparate sources, require strong assumptions on the attribution of hospital lengths of stay and mortality to BSI and assume independence of the cost of managing BSIs and CVC type.

In conclusion, the results of the economic evaluation indicate that replacing standard polyurethane CVCs with antibiotic-impregnated CVCs in PICUs will result in reduced rates of BSI. Given the low background rate of BSI, the variation in costs between CVCs and the sensitivity of the ICER to the time-horizon of analysis, it remains uncertain if antibiotic-impregnated CVCs are cost-effective from a UK NHS perspective. Given the focus of the evaluation, there is limited generalizability outside the UK to other payers, healthcare systems or jurisdictions; however, our economic findings from CATCH add to evidence on the generalizability of trial participants in the UK, and on the cost implications of using antibiotic-impregnated CVCs to the NHS (Harron et al., 2016b).

## Ethics statement

The Research Ethics Committee for South West England approved the study protocol (reference number 09/H0206/69). Prospective written parental consent during preoperative assessment was sought for children admitted to pediatric intensive care units after elective surgery. For children who needed a central venous catheter as an emergency, we sought written parental consent after randomization and stabilization (deferred consent) to avoid delaying treatment. Parents consented to the use of their child's data for the trial, to follow-up using routinely recorded clinical data, and to an additional 0.5 mL of blood being collected for PCR testing whenever a blood culture was clinically needed.

## CATCH trial management group

Ruth Gilbert (chair and chief investigator), Carrol Gamble, Kerry Dwan, Tracy Moitt, Rachel Breen, Colin Ridyard, Angie Wade, Dyfrig Hughes, Quen Mok, Liz Draper, Shane Tibby, Mike Millar, Oliver Bagshaw and Padmanabhan Ramnarayan, Julia Harris and Darren Hewett. Other contributors were Michaela Blundell (quality assurance checks), Susan Howlin and Lynsey Finnetty (data management), and Ivana Pribramska (administrative support).

## Author contributions

DH and RG conceptualized the study; CR, RG, and DH made substantial contribution to the study design and acquisition of data; CR, CP, RG, and DH made substantial contribution to the analysis and interpretation of data, revised the paper critically for important intellectual content and approved the final manuscript.

### Conflict of interest statement

The authors declare that the research was conducted in the absence of any commercial or financial relationships that could be construed as a potential conflict of interest.

## Footnotes

^1^*Health and Social Care Information Centre Data Linkage and Extract Service*. Available online at: http://www.hscic.gov.uk/dles (Accessed August 10, 2017).

^2^*Paediatric Intensive Care Audit Network*. Available online at: http://www.picanet.org.uk/ (Accessed August 10, 2017).

^3^*National Schedule of Reference Costs 2012-13*. Available online at: https://www.gov.uk/government/publications/nhs-reference-costs-2012-to-2013 (Accessed August 10, 2017).
