# Supplementary material for: Cost-Effectiveness of Pediatric Central Venous Catheters in the UK: A Secondary Publication from the CATCH Clinical Trial
Source: Front Pharmacol. 2017 Sep 19;8:644. doi: 10.3389/fphar.2017.00644 (PMC5610787; doi:10.3389/fphar.2017.00644)
Supplement: Supplementary file 1 [file Table1.DOCX]

**Supplementary Appendix Table 1**. A list of all HRGs, costed using the National Tariff guidance.

| **HRG** | **HRG name (inpatient)** | **Elective spell tariff (£)** | **Elective long stay trimpoint (days)** | **Non-elective spell tariff** | **Non-elective long stay trimpoint (days)** | **Per day long stay payment (for days exceeding trimpoint)** |
| --- | --- | --- | --- | --- | --- | --- |
| LB08Z^a^ | Ureter Major Endoscopic Procedure | £1,379.00 | 5 | £2,468.00 | 12 | £301.00 |
| PA03Z^a^ | Febrile Convulsions | £928.00 | 1 | £661.00 | 3 | £304.00 |
| PA14A^a^ | Lower Respiratory Tract Disorders without Acute Bronchiolitis with CC | £3,215.00 | 13 | £2,473.00 | 14 | £222.00 |
| CZ06N^a^ | Minor throat Procedures with CC | £1,431.00 | 3 | £3,222.00 | 24 | £281.00 |
| FZ03B^b^ | Diagnostic and intermediate procedures on the upper GI tract 18 years and under | £852.00 | 5 | £1,267.00 | 5 | £223.00 |
| GB04A^b^ | Endoscopic/Radiology category 1 with Major CC | £1,879.00 | 8 | £6,347.00 | 54 | £228.00 |
| AA16Z | Intracranial Procedures Except Trauma with Non-Transient Stroke or Cerebrovascular Accident, Nervous system infections or Encephalopathy -category 1 or 2 | £4,255.00 | 24 | £7,371.00 | 66 | £210.00 |
| EA24Z | Complex Congenital Surgery | £9,631.00 | 21 | £14,934.00 | 46 | £205.00 |
| EA25Z | Intermediate Congenital Surgery | £9,571.00 | 19 | £13,009.00 | 58 | £205.00 |
| PA14C | Lower Respiratory Tract Disorders without Acute Bronchiolitis with length of stay 1 day or more with CC | £3,602.00 | 22 | £2,301.00 | 15 | £291.00 |
| DZ07B | Fibre optic Bronchoscopy 18 years and under | £1,146.00 | 5 | £1,394.00 | 5 | £190.00 |
| VA11D | Multiple trauma diagnoses, score >=51 with interventions, score 1 - 8 | £5,246.00 | 94 | £5,246.00 | 94 | £232.00 |
| PA16A | Major Infections with CC | £1,719.00 | 8 | £2,856.00 | 22 | £291.00 |
| QZ15B | Therapeutic Endovascular Procedures with Intermediate CC | £1,523.00 | 5 | £5,389.00 | 49 | £227.00 |
| DZ37B | Non-Invasive Ventilation Support Assessment 18 years and under | £927.00 | 5 | £927.00 | 5 | £190.00 |
| PA23B | Cardiac Conditions without CC | £1,420.00 | 5 | £1,427.00 | 5 | £291.00 |
| PA15B | Acute Bronchiolitis without CC | £1,066.00 | 8 | £910.00 | 8 | £291.00 |
| PA23A | Cardiac Conditions with CC | £1,956.00 | 5 | £3,638.00 | 16 | £291.00 |
| EA23Z | Major Complex Congenital Surgery | £12,638.00 | 36 | £19,436.00 | 66 | £205.00 |
| PA14E | Lower Respiratory Tract Disorders without Acute Bronchiolitis with length of stay 0 days | £561.00 | 5 | £434.00 | 5 | £291.00 |
| FZ11A | Large Intestine - Major Procedures with Major CC | £5,441.00 | 33 | £8,053.00 | 68 | £228.00 |
| PA63B | Head, Neck and Ear Disorders with length of stay 1 day or more with CC | £2,514.00 | 9 | £972.00 | 6 | £291.00 |
| PA48A | Blood Cell Disorders with CC | £1,474.00 | 8 | £2,335.00 | 10 | £291.00 |
| PB02Z | Minor Neonatal Diagnoses | £1,041.00 | 14 | £1,041.00 | 14 | £291.00 |
| FZ07A | Major Small Intestine Procedures with CC | £4,569.00 | 19 | £8,028.00 | 62 | £228.00 |
| VA15D | Multiple trauma diagnoses, score >=51 with interventions, score >=45 | £20,844.00 | 143 | £20,844.00 | 143 | £232.00 |
| HC12Z | Intradural Spine Minor 1 | £571.00 | 5 | £739.00 | 5 | £231.00 |
| PA12Z | Asthma or Wheezing | £563.00 | 5 | £622.00 | 5 | £291.00 |
| FZ06A | Very Major Small Intestine Procedures with CC | £7,781.00 | 40 | £8,308.00 | 63 | £228.00 |
| PA25B | Major Gastrointestinal or Metabolic Disorders without CC | £949.00 | 5 | £1,177.00 | 8 | £291.00 |
| AA21Z | Intracranial Procedures Except Trauma with Other Diagnoses - category 1 or 2 | £1,096.00 | 5 | £5,346.00 | 42 | £210.00 |
| HB99Z | Other Procedures for non Trauma | £331.00 | 5 | £331.00 | 5 | £231.00 |
| GA05B | Hepatobiliary Procedures category 5 without CC | £5,598.00 | 17 | £5,980.00 | 35 | £221.00 |
| PA47Z | Sickle-cell Anaemia with Crisis | £494.00 | 8 | £1,587.00 | 9 | £291.00 |
| QZ15A | Therapeutic Endovascular Procedures with Major CC | £9,835.00 | 86 | £10,258.00 | 107 | £227.00 |
| AA02Z | Intracranial Procedures for Trauma with Intermediate Diagnosis | £6,738.00 | 40 | £6,738.00 | 40 | £210.00 |
| EA26Z | Standard Congenital Surgery | £5,615.00 | 15 | £5,615.00 | 15 | £205.00 |
| QZ14A | Vascular Access except for Renal Replacement Therapy with CC | £548.00 | 5 | £1,353.00 | 8 | £227.00 |
| PA02A | Epilepsy Syndrome with CC | £1,043.00 | 5 | £942.00 | 5 | £291.00 |
| PA15A | Acute Bronchiolitis with CC | £2,254.00 | 15 | £1,962.00 | 14 | £291.00 |
| PA25A | Major Gastrointestinal or Metabolic Disorders with CC | £1,715.00 | 5 | £2,583.00 | 14 | £291.00 |
| QZ13A | Vascular Access for Renal Replacement Therapy with CC | £1,287.00 | 5 | £1,571.00 | 8 | £227.00 |
| VA14D | Multiple trauma diagnoses, score >=51 with interventions, score 30 - 44 | £11,259.00 | 129 | £11,259.00 | 129 | £232.00 |
| DZ06Z | Minor Thoracic Procedures | £729.00 | 5 | £1,063.00 | 5 | £190.00 |
| DZ03A | Major Thoracic Procedures with CC | £3,371.00 | 14 | £6,985.00 | 39 | £190.00 |
| AA20Z | Intracranial Procedures Except Trauma with Muscular, Balance, Cranial or Peripheral Nerve disorders or Epilepsy- category 1 or 2 | £1,957.00 | 10 | £3,883.00 | 32 | £210.00 |
| CZ07O | Exteriorisation of Trachea with Major CC | £8,640.00 | 98 | £7,363.00 | 95 | £250.00 |
| PB01Z | Major Neonatal Diagnoses | £1,511.00 | 16 | £1,511.00 | 16 | £291.00 |
| HB16B | Minor Hip Procedures for non Trauma Category 1 with CC | £1,267.00 | 33 | £1,267.00 | 33 | £231.00 |
| FZ05A | Major Stomach or Duodenum Procedures 2 years and over with CC | £3,591.00 | 16 | £6,539.00 | 57 | £228.00 |
| FZ05C | Major Stomach or Duodenum Procedures 1 year and under | £5,402.00 | 16 | £4,582.00 | 11 | £228.00 |
| PA44Z | Neoplasm diagnoses with length of stay 0 days | £541.00 | 5 | £527.00 | 5 | £291.00 |
| FZ12A | General Abdominal - Very Major or Major Procedures with Major CC | £5,070.00 | 29 | £6,963.00 | 54 | £228.00 |
| PA18A | Minor Infections with CC | £843.00 | 5 | £1,204.00 | 8 | £291.00 |
| AA09Z | Intracranial Procedures Except Trauma with Other Diagnoses - category 4 | £2,396.00 | 5 | £8,293.00 | 49 | £210.00 |
| HB63Z | Minor Shoulder and Upper Arm Procedures for non Trauma | £1,401.00 | 5 | £1,401.00 | 5 | £231.00 |
| EA20Z | Other Complex Cardiac Surgery and Re-do's | £10,511.00 | 26 | £12,806.00 | 57 | £205.00 |
| EA14Z | Coronary Artery Bypass Graft (First Time) | £7,358.00 | 16 | £9,055.00 | 39 | £205.00 |
| EA12Z | Implantation of Cardioverter - Defibrillator only | £5,556.00 | 5 | £7,248.00 | 34 | £205.00 |
| DZ02A | Complex Thoracic Procedures with Major CC | £8,271.00 | 31 | £9,426.00 | 54 | £190.00 |
| VA11B | Multiple trauma diagnoses, score 24 - 32 with interventions, score 1 - 8 | £3,864.00 | 24 | £3,864.00 | 24 | £232.00 |
| PA26A | Other Gastrointestinal or Metabolic Disorders with CC | £1,603.00 | 5 | £1,076.00 | 5 | £291.00 |
| PA59C | Major Congenital Conditions under 1 year with CC | £2,444.00 | 8 | £3,609.00 | 31 | £291.00 |
| PA59E | Major Congenital Conditions 1 year and over with CC | £1,148.00 | 5 | £3,142.00 | 13 | £291.00 |
| PA08B | Intermediate Injury without Intracranial Injury without CC | £790.00 | 5 | £757.00 | 5 | £291.00 |
| PA28A | Feeding Difficulties and Vomiting with CC | £2,136.00 | 10 | £1,012.00 | 5 | £291.00 |
| HB23B | Intermediate Knee Procedures for non Trauma with CC | £2,342.00 | 29 | £2,342.00 | 29 | £231.00 |
| PA19B | Viral Infections with length of stay 2 days or more | £1,255.00 | 5 | £1,255.00 | 5 | £291.00 |
| PA06Z | Head Injury with Intracranial Injury | £1,689.00 | 9 | £1,689.00 | 9 | £291.00 |
| PA67Z | Diabetes Mellitus with Ketoacidosis or Coma | £954.00 | 6 | £954.00 | 6 | £291.00 |
| PA45Z | Febrile Neutropenia with Malignancy | £8,858.00 | 51 | £3,894.00 | 13 | £291.00 |
| LB10Z | Bladder Major Open Procedures / Reconstruction | £5,348.00 | 24 | £7,019.00 | 52 | £215.00 |
| PA17A | Intermediate Infections with CC | £1,067.00 | 5 | £1,274.00 | 9 | £291.00 |
| PA03B | Febrile Convulsions 1 year and over | £705.00 | 5 | £595.00 | 5 | £291.00 |
| QZ05A | Miscellaneous Vascular Procedures with CC | £1,687.00 | 5 | £3,733.00 | 30 | £227.00 |
| VA13D | Multiple trauma diagnoses, score >=51 with interventions, score 19 - 29 | £8,858.00 | 117 | £8,858.00 | 117 | £232.00 |
| AA10Z | Intracranial Procedures Except Trauma with Non-Transient Stroke or Cerebrovascular Accident, Nervous system infections or Encephalopathy - category 3 | £7,598.00 | 74 | £11,733.00 | 74 | £210.00 |
| JC01A | Major Multiple Skin Procedures with Major CC | £9,610.00 | 62 | £9,608.00 | 79 | £223.00 |
| GA05A | Hepatobiliary Procedures category 5 with CC | £6,767.00 | 26 | £7,488.00 | 57 | £221.00 |
| EA52Z | Repair or replacement of more than one heart valve | £12,196.00 | 31 | £15,633.00 | 84 | £205.00 |
| DZ03B | Major Thoracic Procedures without CC | £2,429.00 | 9 | £3,884.00 | 20 | £190.00 |
| QZ04Z | Extracranial or Upper Limb Arterial Surgery | £3,567.00 | 7 | £5,606.00 | 34 | £227.00 |
| EA39Z | Pacemaker Procedure without Generator Implant (includes resiting and removal of cardiac pacemaker system) | £2,748.00 | 5 | £5,302.00 | 33 | £205.00 |
| PA38D | Renal Disease with Renal Failure with length of stay 1 day or more | £3,800.00 | 9 | £3,184.00 | 15 | £291.00 |
| PA07B | Head Injury without Intracranial Injury without CC | £539.00 | 5 | £506.00 | 5 | £291.00 |
| VA10D | Multiple trauma diagnoses, score >=51 with no interventions | £3,712.00 | 93 | £3,712.00 | 93 | £232.00 |
| PA21A | Infectious and Non-Infectious Gastroenteritis with CC | £1,855.00 | 8 | £810.00 | 5 | £291.00 |
| PA16B | Major Infections without CC | £696.00 | 5 | £1,659.00 | 11 | £291.00 |
| FZ01C | Complex Oesophageal Procedures 18 years and under | £14,175.00 | 63 | £14,175.00 | 63 | £228.00 |
| QZ01A | Aortic or Abdominal Surgery with CC | £6,487.00 | 27 | £7,307.00 | 53 | £227.00 |
| HB13Z | Intermediate Hip Procedures for non Trauma Category 2 | £5,194.00 | 26 | £5,194.00 | 26 | £231.00 |
| HC08Z | Intradural Spine Major 1 | £4,992.00 | 31 | £4,992.00 | 31 | £231.00 |
| AA11Z | Intracranial Procedures Except Trauma with Haemorrhagic Cerebrovascular Disorders - category 3 | £6,166.00 | 50 | £8,917.00 | 50 | £210.00 |
| PA01A | Nervous System Disorders with CC | £1,146.00 | 5 | £2,368.00 | 15 | £291.00 |
| PA34A | Musculoskeletal or Connective Tissue Disorders with CC | £1,112.00 | 5 | £1,246.00 | 8 | £291.00 |
| LB02D | Kidney Major Open Procedure 18 years and under | £4,289.00 | 7 | £8,972.00 | 36 | £215.00 |
| HB16C | Minor Hip Procedures for non Trauma Category 1 without CC | £969.00 | 5 | £969.00 | 5 | £231.00 |
| VA12D | Multiple trauma diagnoses, score >=51 with interventions, score 9 - 18 | £7,012.00 | 102 | £7,012.00 | 102 | £232.00 |
| CZ01S | Minor Mouth or Throat Procedures 18 years and under with CC | £1,551.00 | 5 | £3,137.00 | 8 | £250.00 |
| HB14B | Intermediate Hip Procedures for non Trauma Category 1 with CC | £3,509.00 | 61 | £3,509.00 | 61 | £231.00 |
| PA63A | Head, Neck and Ear Disorders with length of stay 0 days | £540.00 | 5 | £383.00 | 5 | £291.00 |
| PA60C | Other Congenital Conditions under 1 year with CC | £945.00 | 5 | £1,336.00 | 9 | £291.00 |
| VA10C | Multiple trauma diagnoses, score 33 - 50 with no interventions | £3,453.00 | 47 | £3,453.00 | 47 | £232.00 |
| HB15F | Minor Hip Procedures for non Trauma Category 2 18 years and under with CC | £1,725.00 | 23 | £1,725.00 | 23 | £231.00 |
| FZ20C | Appendicectomy Procedures 18 years and under | £2,367.00 | 5 | £2,292.00 | 7 | £228.00 |
| FZ27D | Endoscopic or Intermediate General Abdominal Procedures 18 years and under | £1,216.00 | 5 | £1,729.00 | 8 | £228.00 |
| LA05Z | Renal Replacement Peritoneal Dialysis Associated Procedures | £1,138.00 | 5 | £1,195.00 | 5 | £215.00 |
| LB05D | Kidney Intermediate, Endoscopic and Percutaneous Interventions 18 years and under | £2,372.00 | 5 | £4,973.00 | 25 | £215.00 |
| AA04Z | Intracranial Procedures Except Trauma with Non-Transient Stroke or Cerebrovascular Accident, Nervous system infections or Encephalopathy-category 4 | £7,936.00 | 74 | £11,733.00 | 74 | £210.00 |
| PA19A | Viral Infections with length of stay 1 day or less | £446.00 | 5 | £444.00 | 5 | £291.00 |
| PA21B | Infectious and Non-Infectious Gastroenteritis without CC | £705.00 | 5 | £520.00 | 5 | £291.00 |
| EA11Z | Percutaneous Congenital Interventions: Other including Septostomy, Embolisations, Non-coronary Stents and Energy Moderated Perforation | £1,934.00 | 5 | £4,417.00 | 33 | £205.00 |
| FZ25B | Therapeutic Endoscopic or Intermediate Stomach or Duodenum Procedures 18 years and under | £996.00 | 5 | £996.00 | 5 | £228.00 |
| FZ04A | Very Major Stomach or Duodenum Procedures with Major CC | £8,135.00 | 44 | £11,299.00 | 84 | £228.00 |
| AB04Z | Major Pain Procedures | £570.00 | 5 | £2,624.00 | 24 | £210.00 |
| LB11B | Urinary Diversion without Cystectomy without Malignancy | £5,913.00 | 30 | £9,562.00 | 71 | £215.00 |
| VA12C | Multiple trauma diagnoses, score 33 - 50 with interventions, score 9 - 18 | £6,487.00 | 61 | £6,487.00 | 61 | £232.00 |
| VA11A | Multiple trauma diagnoses, score <=23 with interventions, score 1 - 8 | £1,965.00 | 10 | £1,965.00 | 10 | £232.00 |
| SA14Z | Plasma Exchanges 2 to 9 | £2,385.00 | 13 | £7,293.00 | 55 | £237.00 |
| AA19Z | Intracranial Procedures Except Trauma with Cerebral Degenerations or Miscellaneous Disorders of Nervous System - category 1 or 2 | £1,843.00 | 8 | £5,014.00 | 25 | £210.00 |
| VA11C | Multiple trauma diagnoses, score 33 - 50 with interventions, score 1 - 8 | £4,513.00 | 45 | £4,513.00 | 45 | £232.00 |
| PB03Z | Healthy Baby | £0.00 | 5 | £0.00 | 5 | £0.00 |
| PA49Z | Coagulation Disorders | £666.00 | 5 | £691.00 | 5 | £291.00 |
| PA35A | Skin Disorders with CC | £1,103.00 | 5 | £1,187.00 | 8 | £291.00 |
| PA42Z | Brain Tumours with length of stay 1 day or more | £2,660.00 | 9 | £2,830.00 | 9 | £291.00 |
| GB03A | Endoscopic/Radiology category 2 with CC | £1,020.00 | 5 | £6,235.00 | 53 | £221.00 |
| GB01Z | Endoscopic/Radiology category 4 | £2,366.00 | 9 | £4,813.00 | 58 | £221.00 |
| PA68Z | Diabetes Mellitus without Ketoacidosis or Coma | £941.00 | 5 | £941.00 | 5 | £291.00 |
| QZ15C | Therapeutic Endovascular Procedures without CC | £1,178.00 | 5 | £3,091.00 | 26 | £227.00 |
| SA15Z | Plasma Exchanges 10 to 19 | £4,892.00 | 13 | £7,293.00 | 55 | £237.00 |
| PA51Z | Child Safeguarding (welfare and protection) | £854.00 | 5 | £854.00 | 5 | £291.00 |
| GA03A | Hepatobiliary Procedures category 7 with CC | £10,784.00 | 49 | £14,235.00 | 71 | £221.00 |
| DZ02B | Complex Thoracic Procedures with CC | £6,356.00 | 18 | £6,729.00 | 26 | £190.00 |
| PA14D | Lower Respiratory Tract Disorders without Acute Bronchiolitis with length of stay 1 day or more without CC | £2,286.00 | 16 | £1,035.00 | 6 | £291.00 |
| CZ04Q | Complex Major Mouth or Throat Procedures without CC | £7,565.00 | 39 | £7,565.00 | 39 | £250.00 |
| AA17Z | Intracranial Procedures Except Trauma with Haemorrhagic Cerebrovascular Disorders - category 1 or 2 | £2,794.00 | 12 | £6,852.00 | 64 | £210.00 |
| QZ11B | Amputations without Major CC | £8,011.00 | 53 | £10,771.00 | 95 | £227.00 |
| HC11Z | Intradural Spine Minor 2 | £3,138.00 | 18 | £3,138.00 | 18 | £231.00 |
| EA10Z | Percutaneous Congenital Interventions: Balloon valve intermediate interventions and arterial duct closure | £4,111.00 | 6 | £8,275.00 | 48 | £205.00 |
| FZ02Z | Very Major Oesophageal Procedures | £3,802.00 | 11 | £6,158.00 | 39 | £228.00 |
| FZ11B | Large Intestine - Major Procedures without Major CC | £2,643.00 | 14 | £4,640.00 | 32 | £228.00 |
| AA15Z | Intracranial Procedures Except Trauma with Other Diagnoses - category 3 | £2,396.00 | 5 | £8,293.00 | 49 | £210.00 |
| QZ05B | Miscellaneous Vascular Procedures without CC | £1,035.00 | 5 | £2,402.00 | 14 | £227.00 |
| EA43Z | Implantation of Prosthetic Heart or Ventricular Assist Device | £42,583.00 | 90 | £42,583.00 | 90 | £205.00 |
| FZ07B | Major Small Intestine Procedures without CC | £3,134.00 | 15 | £4,551.00 | 28 | £228.00 |
