# Supplementary material for: Cost-Effectiveness of Pediatric Central Venous Catheters in the UK: A Secondary Publication from the CATCH Clinical Trial
Source: Front Pharmacol. 2017 Sep 19;8:644. doi: 10.3389/fphar.2017.00644 (PMC5610787; doi:10.3389/fphar.2017.00644)
Supplement: Supplementary file 2 [file Table2.DOCX]

**Supplementary Appendix Table 2**. A list of all HRGs, associated with outpatient attendances.

| **Treatment function** | **Treatment function name (outpatient)** | **WF01B *First Attendance - Single Professional*** | **WF02B *First Attendance - Multi Professional*** | **WF01A *Follow Up Attendance - Single Professional*** | **WF02A *Follow Up Attendance - Multi Professional*** |
| --- | --- | --- | --- | --- | --- |
| 100 | General Surgery | £191.00 | £207.00 | £101.00 | £101.00 |
| 101 | Urology | £177.00 | £196.00 | £96.00 | £99.00 |
| 103 | Breast Surgery | £154.00 | £154.00 | £84.00 | £85.00 |
| 104 | Colorectal Surgery | £131.00 | £157.00 | £72.00 | £105.00 |
| 105 | Hepatobiliary & Pancreatic Surgery | £166.00 | £166.00 | £102.00 | £102.00 |
| 106 | Upper Gastrointestinal Surgery | £140.00 | £140.00 | £82.00 | £82.00 |
| 107 | Vascular Surgery | £234.00 | £234.00 | £116.00 | £116.00 |
| 110 | Trauma & Orthopaedics | £137.00 | £137.00 | £83.00 | £83.00 |
| 120 | ENT | £114.00 | £141.00 | £63.00 | £73.00 |
| 130 | Ophthalmology | £115.00 | £138.00 | £67.00 | £75.00 |
| 140 | Oral Surgery | £130.00 | £185.00 | £80.00 | £126.00 |
| 143 | Orthodontics | £186.00 | £285.00 | £83.00 | £129.00 |
| 144 | Maxillo-Facial Surgery | £115.00 | £190.00 | £70.00 | £99.00 |
| 160 | Plastic Surgery | £117.00 | £131.00 | £67.00 | £85.00 |
| 170 | Cardiothoracic Surgery | £227.00 | £227.00 | £142.00 | £162.00 |
| 171 | Paediatric Surgery | £191.00 | £241.00 | £109.00 | £163.00 |
| 172 | Cardiac Surgery | £293.00 | £293.00 | £171.00 | £171.00 |
| 173 | Thoracic Surgery | £260.00 | £260.00 | £159.00 | £159.00 |
| 190 | Anaesthetics | £98.00 | £98.00 | £95.00 | £95.00 |
| 191 | Pain Management | £181.00 | £195.00 | £91.00 | £119.00 |
| 211 | Paediatric Urology | £182.00 | £196.00 | £111.00 | £111.00 |
| 214 | Paediatric Trauma & Orthopaedics | £154.00 | £163.00 | £100.00 | £113.00 |
| 215 | Paediatric Ear Nose & Throat | £116.00 | £141.00 | £74.00 | £74.00 |
| 216 | Paediatric Ophthalmology | £156.00 | £172.00 | £89.00 | £125.00 |
| 217 | Paediatric Maxillo-Facial Surgery | £154.00 | £190.00 | £116.00 | £116.00 |
| 219 | Paediatric Plastic Surgery | £182.00 | £182.00 | £98.00 | £98.00 |
| 251 | Paediatric Gastroenterology | £279.00 | £279.00 | £158.00 | £158.00 |
| 252 | Paediatric Endocrinology | £305.00 | £352.00 | £172.00 | £185.00 |
| 253 | Paediatric Clinical Haematology | £414.00 | £464.00 | £218.00 | £247.00 |
| 257 | Paediatric Dermatology | £149.00 | £168.00 | £107.00 | £108.00 |
| 258 | Paediatric Respiratory Medicine | £315.00 | £315.00 | £172.00 | £172.00 |
| 263 | Paediatric Diabetic Medicine | £353.00 | £353.00 | £119.00 | £119.00 |
| 300 | General Medicine | £210.00 | £251.00 | £105.00 | £121.00 |
| 301 | Gastroenterology | £265.00 | £265.00 | £83.00 | £116.00 |
| 302 | Endocrinology | £230.00 | £230.00 | £106.00 | £116.00 |
| 303 | Clinical Haematology | £268.00 | £288.00 | £106.00 | £106.00 |
| 306 | Hepatology | £224.00 | £290.00 | £139.00 | £151.00 |
| 307 | Diabetic Medicine | £242.00 | £321.00 | £99.00 | £147.00 |
| 320 | Cardiology | £210.00 | £251.00 | £105.00 | £121.00 |
| 321 | Paediatric Cardiology | £289.00 | £289.00 | £170.00 | £170.00 |
| 329 | Transient Ischaemic Attack | £477.00 | £477.00 | - | - |
| 330 | Dermatology | £112.00 | £168.00 | £69.00 | £108.00 |
| 340 | Respiratory Medicine | £223.00 | £244.00 | £105.00 | £128.00 |
| 341 | Respiratory Physiology | £189.00 | £189.00 | £122.00 | £122.00 |
| 350 | Infectious Diseases | £255.00 | £255.00 | £195.00 | £195.00 |
| 360 | Genito-Urinary Medicine | £133.00 | £148.00 | £82.00 | £82.00 |
| 361 | Nephrology | £299.00 | £454.00 | £124.00 | £219.00 |
| 370 | Medical Oncology | £228.00 | £290.00 | £98.00 | £115.00 |
| 410 | Rheumatology | £246.00 | £246.00 | £102.00 | £102.00 |
| 420 | Paediatrics | £231.00 | £288.00 | £129.00 | £159.00 |
| 430 | Geriatric Medicine | £303.00 | £303.00 | £139.00 | £139.00 |
| 501 | Obstetrics | £119.00 | £154.00 | £60.00 | £60.00 |
| 502 | Gynaecology | £138.00 | £142.00 | £81.00 | £99.00 |
| 503 | Gynaecological Oncology | £154.00 | £271.00 | £90.00 | £132.00 |
| 560 | Midwife Episode | £119.00 | £154.00 | £60.00 | £60.00 |
| 800 | Clinical Oncology | £228.00 | £290.00 | £98.00 | £115.00 |
| 812 | Diagnostic Imaging | £0.00 | £0.00 | £0.00 | £0.00 |
