# Supplementary material for: Cost-Effectiveness of Pediatric Central Venous Catheters in the UK: A Secondary Publication from the CATCH Clinical Trial
Source: Front Pharmacol. 2017 Sep 19;8:644. doi: 10.3389/fphar.2017.00644 (PMC5610787; doi:10.3389/fphar.2017.00644)
Supplement: Supplementary file 3 [file Table3.DOCX]

**Supplementary Appendix Table 3**. A list of all HRGs associated with accident and emergency (A&E) attendances.

| **HRG code** | **HRG name (A&E)** | **Band** | **24 hour A&E units tariff (£)** | **Non-24 hour A&E units and MIUs tariff (£)** |
| --- | --- | --- | --- | --- |
| VB01Z | Any investigation with category 5 treatment | 1 | £235.00 | £54.00 |
| VB02Z | Category 3 investigation with category 4 treatment | 1 | £235.00 | £54.00 |
| VB03Z | Category 3 investigation with category 1-3 treatment | 2 | £151.00 | £54.00 |
| VB04Z | Category 2 investigation with category 4 treatment | 2 | £151.00 | £54.00 |
| VB05Z | Category 2 investigation with category 3 treatment | 2 | £151.00 | £54.00 |
| VB06Z | Category 1 investigation with category 3-4 treatment | 3 | £81.00 | £54.00 |
| VB07Z | Category 2 investigation with category 2 treatment | 4 | £112.00 | £54.00 |
| VB08Z | Category 2 investigation with category 1 treatment | 4 | £112.00 | £54.00 |
| VB09Z | Category 1 investigation with category 1-2 treatment | 3 | £81.00 | £54.00 |
| VB10Z | Dental Care | 5 | £54.00 | £54.00 |
| VB11Z | No investigation with no significant treatment | 5 | £54.00 | £54.00 |

Notes: ^a^2010-11 tariffs, ^b^2011-12 tariff; otherwise 2012-13 tariff
