# Supplementary material for: Cost-Effectiveness of Pediatric Central Venous Catheters in the UK: A Secondary Publication from the CATCH Clinical Trial
Source: Front Pharmacol. 2017 Sep 19;8:644. doi: 10.3389/fphar.2017.00644 (PMC5610787; doi:10.3389/fphar.2017.00644)
Supplement: Supplementary file 4 [file Table4.DOCX]

**Supplementary Appendix Table 4**. Hospital ward bed-day rates as provided by hospital finance departments and adjusted for inflation (£ sterling, 2013)

| **Hospital** | **HES hospital ID** | **Market Forces Factor^a^** | **Ward Rate^b^** |
| --- | --- | --- | --- |
| Birmingham Children's Hospital | RQ3 | 1.05 | £289.60 |
| Bristol Royal Hospital for Children | RA7 | 1.08 | £366.00 |
| Evelina Children's Hospital | RJ1 | 1.28 | £595.35^c^ |
| Freeman Hospital | RTD | 1.04 | £595.35^c^ |
| Alder Hey | RBS | 1.04 | £364.00^d^ |
| Glenfield Hospital | RWE | 1.04 | £751.04 |
| Great Ormond Street Hospital | RP4 | 1.29 | £2,156.70 |
| Leeds General Infirmary | RR8 | 1.05 | £542.01 |
| Leicester Royal Infirmary | RWE | 1.04 | £751.04 |
| Queens Medical Centre | RX1 | 1.04 | £374.31 |
| Royal Brompton Hospital | RT3 | 1.25 | £369.72 |
| Royal Victoria Infirmary | RTD | 1.25 | £342.00 |
| Southampton General Hospital | RHM | 1.09 | £212.11 |
| St Mary's | RYJ | 1.24 | £394.37 |

^a^used with HRGs only; ^b^ ward rate excludes ICU or HDU costs; ^c^ mean of series of wards provided by all hospitals except Alder Hey; ^d^ mean of series of wards provided by hospital
